# Specificity of serum prostate-specific antigen determination in the Finnish prostate cancer screening trial

**DOI:** 10.1038/sj.bjc.6603522

**Published:** 2007-01-09

**Authors:** L Määttänen, M Hakama, T L J Tammela, M Ruutu, M Ala-Opas, H Juusela, P Martikainen, U-H Stenman, A Auvinen

**Affiliations:** 1 Finnish Cancer Registry, Liisankatu 21 B, FIN-00170 Helsinki, Finland; 2 Tampere School of Public Health, FIN-33014 University of Tampere, Tampere, Finland; 3 Department of Urology, Tampere University Hospital and University of Tampere, Box 2000, FIN-33521 Tampere, Finland; 4 Department of Urology, Helsinki University Hospital, Box 580 FIN-00029, Helsinki, Finland; 5 Department of Surgery, Jorvi Hospital, Turuntie 150, FIN-02740 Espoo, Finland; 6 Department of Pathology, Tampere University Hospital, University of Tampere, Box 2000, FIN-33521 Tampere, Finland; 7 Department of Clinical Chemistry, Helsinki University Hospital, Box 700, FIN-00029 Helsinki, Finland; 8 Finnish Cancer Institute, Liisankatu 21 B, FIN-00170 Helsinki, Finland

**Keywords:** prostate neoplasms, mass screening, specificity, randomised controlled trial, prostate-specific antigen

## Abstract

Specificity constitutes a component of validity for a screening test. The number of false-positive (FP) results has been regarded as one of major shortcomings in prostate cancer screening. We estimated the specificity of serum prostate-specific antigen (PSA) determination in prostate cancer screening using data from a randomised, controlled screening trial conducted in Finland with 32 000 men in the screening arm. We calculated the specificity as the proportion of men with negative findings (screen negatives, SN) relative to those with negative and FP results (SN/(SN+FP)). A SN finding was defined as either PSA⩽4 ng ml^−1^ or PSA 3.0–3.9 ng ml^−1^ combined with a negative ancillary test (digital rectal examination, DRE or free/total, F/T PSA ratio). False positives were those with positive screening test followed by a negative diagnostic examination. Of the 30 194 eligible men, 20 794 (69%) attended the first screening round and 1968 (9.5%) had a screen-positive finding. A total of 508 prostate cancers were detected at screening (2.4%). Hence, the number of SN findings was 18 825 and the number of FP results 1358. Specificity was estimated as 0.933 (18 825 out of 20 183) with 95% confidence interval (CI) 0.929–0.936. Specificity decreased with age. Digital rectal examination as ancillary examination had similar or higher specificity than F/T PSA. In the second screening round, specificity was slightly lower (0.912, 95% CI 0.908–0.916). The specificity of PSA screening in the Finnish screening trial is acceptable. Further improvement in specificity could, however, improve acceptability of screening and decrease screening costs.

The validity of a screening test is the capability to discriminate between those with and without disease, and can be measured by sensitivity and specificity (Hakama, 1991). Ideally, a screening test should be able to classify correctly both subjects with and without the target disorder. In practice, the distributions of test values between these two populations always overlap. Sensitivity indicates the capacity to find persons with disease, whereas specificity is the ability to identify those free of the target disorder. Sensitivity and specificity are characteristics of the test that are independent of the occurrence of the disease in the target population, but may depend on disease characteristics. Specificity is inversely proportional to the frequency of FP tests in those free of disease. In the context of cancer screening, optimal specificity depends on how many negative (unnecessary) biopsies one is willing to accept in order to detect one case of cancer. Specificity of a screening test is an indicator of the adverse effects of screening, including the cost and inconvenience owing to the diagnostic examination. Specificity is a characteristic of the test and an indicator of test performance used in evaluation of screening methods, but not directly applicable in decision making at the individual level.

Prostate cancer is one of the most common cancers among men in the industrial countries (Parkin *et al*, 2002). Serum prostate-specific antigen (PSA) was identified in the 1970s (Ablin *et al*, 1970; Li and Beling 1973) and later shown to be a marker of prostate cancer (Wang *et al*, 1981). It has been adopted for case finding among asymptomatic men, which has substantially increased the detection and incidence of prostate cancer (Hankey *et al*, 1999; Etzioni *et al*, 2002). Opportunistic screening with PSA is widespread, but the evidence for its effectiveness in terms of mortality reduction is still lacking (Auvinen *et al*, 2002). One of the problems with PSA screening is the large proportion of FP results, as PSA is an organ-specific, but not disease-specific marker (Stenman *et al*, 2000). The main cause of elevated serum PSA concentration is benign prostatic hyperplasia. A positive screening test in the absence of disease leads to unnecessary biopsies and constitutes an adverse effect of screening, which adds costs, increases overdiagnosis and overtreatment and can affect acceptability of screening, that is, reduce participation at subsequent screening rounds. This is especially important in population screening, where the proportion of those with disease is low.

The aim of the study was to estimate the specificity of the PSA test in the Finnish prostate cancer screening trial.

## MATERIALS AND METHODS

The Finnish prostate cancer screening trial is the largest centre of the European Randomised Study of Prostate Cancer screening (de Koning *et al*, 2002). It was started in 1996 in two metropolitan regions, Helsinki and Tampere. The study population of 80 458 men at ages 55–67 years was identified from the Population Register Centre of Finland. Men who had denied the use of their addresses were ineligible (approximately 1%), as well as men with a previous prostate cancer (*N*=161) and they were excluded from the trial. During 1996–1999, 8000 men were annually randomly allocated to the screening arm using a computer algorithm based on random numbers and were invited for the first screening round. The second screening round was carried out after a 4-year interval between 2000 and 2003. The rest of the target population comprised the control arm of the trial. Individuals in the control arm were not contacted.

An invitation letter was sent to the men of the screening arm with an information leaflet describing the trial, appended with a brief questionnaire about urological symptoms, family history of prostate cancer, previous PSA tests and an informed consent form to be signed by the subject. This approach is called randomisation before consent or Zelen-type randomisation (Zelen, 1979).

After an informed consent, a blood sample was drawn at the local cancer society clinics in Helsinki and Tampere. Serum PSA concentrations were analysed at the Central Laboratory of Helsinki University Hospital by Hybritech Tandem-E for determination of total PSA and Wallac AutoDelfia for free PSA.

Men with serum PSA⩾4 ng ml^−1^ were referred to the local hospital for diagnostic examinations, including three examinations for all men: digital rectal examination (DRE), transrectal ultrasound (TRUS) and prostate biopsy (compliance with biopsy 95%). Men with serum PSA concentration 3.0–3.9 ng ml^−1^ were referred for supplementary test: DRE during the first 3 years and the proportion of free PSA (F/T-PSA) since 1999 with a cutoff point of 0.16. Those with a positive ancillary test were also referred to diagnostic work-up. Diagnosis of prostate cancer was based on histological confirmation. The biopsy protocol consisted initially of sextant biopsies, but the number of cores was increased to 10–12 in 2002. A re-biopsy was carried out if either the PSA was above 10 ng ml^−1^ or the initial histopathologic diagnosis was prostatic intraepithelial neoplasia, atypical small acinar proliferation or unconfirmed suspicion for carcinoma. Information on screen-detected cases was obtained from the trial database and interval cancers were identified from the population-based, nationwide Finnish Cancer Registry (Teppo *et al*, 1994).

Specificity was defined as the proportion of the disease-free men correctly classified as negative by the test (PSA<4.0 mg ml^−1^, or PSA 3.0–3.9 ng ml^−1^ with a positive ancillary test), among men classified as disease-free during the screening episode (including both SN men and those who were screen positive but had a negative biopsy). Confidence interval (CI) for specificity was calculated based on standard error for a proportion, s.e. (p)=√(pq/n).

The study protocol was approved by the ethical committees in each participating hospital. Permission to use the data of the cancer registry was obtained from the Research and Development Center for Welfare and Health (STAKES).

## RESULTS

In the target population of 80 458 men, 8000 men were randomly allocated to the screening arm each year during the enrolment period, 1996–1999. At the time of the invitation to the first screening round, 30 194 were eligible and invited. Of them, 20 794 (69%) participated in the first screening round. A drop-out analysis showed that young age and residence in Helsinki area were associated with non-participation (mean ages 59.8 *vs* 60.2 years and 81% *vs* 72% resident in Helsinki region among non-participants and participants). Of the participants, 18,825 had serum PSA concentration below 3 ng ml^−1^ or PSA 3.0–3.9 ng ml^−1^ in combination with negative DRE or free/total ratio ⩾0.16 (Table 1). Thus, the number of screen-positive men was 1968 and prostate cancer was histologically confirmed in 508 subjects. The histological finding was benign in 1358 men and 102 subjects (5.2%) did not undergo biopsy.

Based on these observations, the specificity of the screening test was estimated as (18 825)/(18 825+1358) that is, 0.933 with 95% CI 0.929–0.936 (Table 2). Men without biopsy were excluded from this calculation. Specificity decreased with increasing age, from 0.97 at age 55 to 0.88 at 67 years.

Assuming a similar proportion of cancers and benign findings among the 102 men who were not biopsied as among the screen-positive men who underwent biopsy (102•(508/{508+1,358})), the number of cancers was estimated as 28 and number of men with FP screening test as 74. Therefore, a corrected estimate of the relative specificity of the PSA test was virtually identical to the original: (18 825)/(18 825+1432)=0.929 (95% CI 0.926–0.933).

After the first 3 years of screening, DRE was replaced with F/T PSA ratio as ancillary test among men with PSA 3.0–3.9 ng ml^−1^. The proportion of FP results among men with PSA in this range was 7% with DRE (59 out of 794) and 16% with F/T PSA ratio >0.16 (44 out of 269). The overall number of SN findings during the three initial years was 14 149 and the number of FP findings 995 (7.0%). During the last year of the first screening round, the corresponding figures were 4540 and 368 (8.1%). Hence, adoption of the F/T PSA to replace DRE was associated with a nonsignificant decrease in specificity of the screening programme, 0.934 (0.930–0.938) *vs* 0.925 (0.917–0.932) in the first screening round.

**Figure 1:**
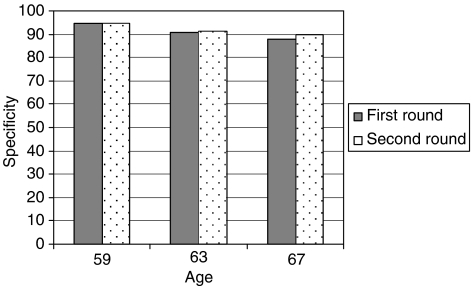
Specificity by age and screening round.

Specificity in the second round was slightly lower compared with the first round (Table 3). A total of 18 612 men were screened and 2303 screen-positive subjects referred to biopsy. Of them, 2156 were biopsied within the study (at the screening centres) and 583 cancers (3.1%) detected. Overall, specificity was 0.912 (95% CI 0.908–0.916, Table 4). Correction for missing biopsy results did not materially affect the estimate (corrected specificity 0.910, 95% CI 0.906–0.914). In men who attended screening for the first time (i.e., were non-participants in the first round), specificity was 0.903 (95% CI 0.891–0.914), with a corrected estimate of 0.911 (0.900–0.921). Similar to the first screening round, specificity decreased with age. However, no obvious difference in specificity was found within age group, that is, when comparing men at the same age in the first *vs* second round. Specificity in the two screening rounds remained comparable (0.917 and 0.922) after restricting the analysis to the three age groups targeted in both rounds (59, 63 and 67 years, Figure 1). This suggests that the decrease in sensitivity between the screening rounds was due to the older age structure alone.

Some alternative screening algorithms can also be evaluated, based on number of screen-positive findings. Had a cutoff limit of 3 ng ml^−1^ been used, the number of test-positive men would have been increased from 1980 (9.5%) to 2762 (13.3%) in the first screening round. For the second screening round, the number of screen-positive tests with a cutoff level of 3 ng ml^−1^ would have increased from 2303 to 3401 compared with the current protocol (12.3% *vs* 18.3% screen positive).

Age-specific cutoff levels (3.5 ng ml^−1^ for ages 55–59 and 4.5 ng ml^−1^ for 63–67) would have resulted in 287 fewer screen-positive findings (from 1980 to 1693, i.e. from 9.5 to 8.1%) in the first screening round, that is, slightly lower compared with the current screening protocol. The number of screen-positive results would have increased for men in their fifties and decreased for older men. In the second screening round with 4 years older subjects, age-specific cutoff levels would have increased the number of screen-positive findings by 557 compared with the protocol used in the trial (from 2303 to 2860 i.e. from 12.4 to 15.4%).

## DISCUSSION

We report a systematic assessment of specificity in relation to its several possible determinants in a population-based trial. Our results show that a reasonably high specificity (above 90%) can be achieved with the PSA test in prostate cancer screening. Moreover, specificity decreases only slightly at repeat (incidence) screening, and this is entirely attributable to ageing of the study subjects.

Overall, specificity of serum PSA as screening test for prostate cancer was slightly above 90%. A Canadian screening study with a cutoff of 3 ng ml^−1^ reported 90% specificity (Labrie *et al*, 1992) and similar findings were reported from the US (Mettlin *et al*, 1994). A volunteer-based study in the US reported specificity of 73% (Punglia *et al*, 2005). A meta-analysis estimated specificity of PSA as 93% at 4.0 ng ml^−1^ (Mistry and Cable, 2003).

Our study population may represent relatively low-risk men, as the trial is population-based and the subjects are fairly young. Yet, the incidence of prostate cancer in Finland is rather high in international comparison, with age-standardised incidence of 84 per 100 000 in 2002 (Ferlay *et al*, 2004). Owing to the representative study population, our findings are likely to be more applicable to the general population than those from volunteer-based studies. Furthermore, we used a consistent definition of specificity, with systematic evaluation of various factors affecting specificity within the screening trial.

Specificity was only slightly lower in the second screening round compared with the first. This was due to participants being older at the second round. The main factor is probably the strong increase in prevalence of benign prostatic hyperplasia with age. Introduction of a new biopsy regimen with increased number of cores may have also decreased the number of apparent FP screening findings (if a larger proportion of true-positive findings were detected). In both rounds, the specificity was higher in the young age groups. This finding indicates that specificity is likely to decrease at subsequent screening rounds, as age at screening increases.

Digital rectal examination as an ancillary test among men with intermediate PSA levels was associated with a lower rate of FP findings than F/T PSA and hence, slightly higher specificity. The yield was also lower than with free PSA (2.1% *vs* 5.2% of men with PSA 3.0–3.9 ng ml^−1^). This is consistent with the findings from a Dutch screening trial, where the specificity of DRE was 91% (Schröder *et al*, 1998). However, the costs for a DRE are substantially higher than determination of F/T PSA in our trial, where a blood sample is drawn initially and can be used for determination of both total and free PSA, whereas DRE requires a separate visit for an urologist.

We estimated the specificity first by assuming that the proportion of false negatives (cancers among SN men surfacing during the screening interval) is negligible and can be ignored. This cross-sectional approach gives a measure that can be called relative specificity. Longitudinal analysis with correction for false-negative results (interval cases) is able to take into account the fact that many men with a negative biopsy do in fact harbour a latent cancer. Yet, adjustment for this did not materially affect the results. However, if all men harbouring a focal carcinoma in their prostates were classified as false negative, the situation would change dramatically as this has been very common in studies based on autopsy (Breslow *et al*, 1977; Kabalin *et al*, 1989) and cystoprostatectomy specimens or prostate tissue removed in transurethral prostatectomy (Montie *et al*, 1989; Merrill and Wiggins, 2002). Studies based on natural history models have estimated that up to 45% of screen-detected cases may be due to overdiagnosis, that is, cancers that would not have surfaced clinically during the man's lifetime if unscreened (Etzioni *et al*, 2002; Draisma *et al*, 2003). Thus, latent or minimal disease is very frequent, and there are good grounds to argue that presence of malignant histological features alone does not constitute a true golden standard for clinically significant prostate cancer. This issue can also been as a problem of FP findings, if overdiagnosed cases (if identifiable) were to be classified as FP findings. Yet, they cannot be reliably identified by current means, even if the above argument was accepted. Both issues, however, emphasise the need for definition of diagnosis of prostate cancer. We have used the conventional approach, but taking into the above uncertainties would have reduced the estimates of specificity.

Not all men with screen-positive result attend diagnostic examinations, and the results may not be available, if medical care is sought outside the screening organisation. In our material, approximately 0.5% of all participants or 5% of screen-positive men did not undergo biopsy within the trial (in the study hospitals). In the screening programme, these men are classified as negatives, that is, no further procedures are undertaken (despite indications being fulfilled). This is problematic when evaluating a screening test. In calculation of specificity, these men were assumed to be true positives and FPs in the same proportion as those biopsied. Owing to the small number of such cases, this did not affect our estimate of test specificity.

No consensus has been established as to the optimal use of PSA and several approaches have been proposed, including age-specific cutoffs and PSA relative to prostate volume (Gretzer and Partin 2003). Cutoff values even lower than 4 ng ml^−1^ have been proposed and are being used in some screening projects (Labrie *et al*, 1992; Krumholtz *et al*, 2002; Punglia *et al*, 2005). In the European Randomized Study of Screening for Prostate Cancer, ERSPC, a cutoff level of 3 ng ml^−1^ instead of 4 ng ml^−1^ was associated with increase in the proportion of test-positive findings from 1.6 to 5.1% (de Koning *et al*, 2002). Generally, both the proportion of screening-positive findings and detection rates have been higher in studies with combined modality screening (e.g., DRE and/or TRUS in addition to PSA). In our study, a limit of 3 ng ml^−1^ would have resulted in an increase in FP tests by more than a third. As the increase in screen-positive findings would be in the low PSA range, where prostate cancer prevalence is likely to be low and FP results more common than at higher PSA levels, adopting a lower cutoff level is likely to reduce specificity.

Age-specific cutoff values have been proposed for PSA in order to improve specificity of the test among older men (Oesterling *et al*, 1993). The rationale is that the prostate volume and prevalence of benign prostatic hyperplasia increase rapidly after 60 years of age. Use of age-specific cutoff levels would have resulted in a similar number of screen-positive findings in the first round, but substantially higher numbers in the second screening round. As no referrals or biopsy decision were made based on the age-specific cutoff values, we were not able to directly assess the possible effect on specificity. It would have resulted in large numbers of screen-positive men in older age groups and lower numbers in younger age groups. Because specificity was inversely correlated with age, it is likely that use of age-specific cutoff values would have resulted in lower specificity.

There are two approaches for avoiding information bias owing to PSA-driven biopsy in assessment of validity of the PSA test. First, it can be argued that everybody should receive the diagnostic test (prostate biopsy) when evaluating specificity, in order to completely identify those with disease. In some studies, all men have been biopsied, regardless of PSA result, which has resulted in detection of prostate cancer even at low PSA levels (Labrie *et al*, 1992; Thompson *et al*, 2004). These studies have also shown similar specificity for PSA as others (90–94%). Alternatively, the distortion from ‘affirming the consequent’ can be avoided, when no test results are followed by diagnostic examination (Walter, 1999). In serum bank studies, the PSA has been determined only afterwards and therefore it has not affected the diagnosis (Gann *et al*, 1995; Hakama *et al*, 2001). Specificity in this context has been estimated as 91–94%. Furthermore, cases in the serum bank studies have been diagnosed mainly before the PSA screening era and also therefore likely to avoid overdiagnosis.

In comparison with screening for other cancers, our results indicate similar or slightly lower specificity for PSA in prostate cancer screening. In mammography screening for breast cancer, specificity has ranged 82–99%, being commonly slightly above 90% (Elmore *et al*, 2005). Fairly similar figures (86–100%) have been reported for the cervical smear in cervix cancer screening (Nanda *et al*, 2000; Cervix cancer screening 2005). In faecal occult blood testing for colorectal cancer, slightly higher specificity (95% or higher) has been found (Allison *et al*, 1996; Rozen *et al*, 2000).

We conclude that screening for prostate cancer based on PSA determination has acceptable specificity. It should, however, be further improved if such screening is to be adopted as public health policy. We do not recommend PSA screening before the results in terms of mortality from prostate cancer are known.

## Figures and Tables

**Table 1:** Number of men by screening result and prostate cancer diagnosis in the first screening round, Finnish prostate cancer screening trial

	**Prostate cancer diagnosis**		
**Screening result**	**Yes**	**No**	**Total**
Positive	508^a^	1358	1866^b^
Negative	42^c^	18 783	18 825
Total	550	20 141	20 691^b^

a Screen-detected prostate cancer.

b A total of 102 screen-positive men failed to undergo biopsy and were excluded.

c Interval cancer among screen-negative men.

**Table 2:** The frequency of TN and FP screening findings by age in the first screening round of the Finnish prostate cancer screening trial

**Age**	**TN^a^**	**FP^b^**	**Specificity (95% CI)^c^**
55	6153	214	0.966 (0.962–0.971)
59	5137	279	0.948 (0.943–0.954)
63	4146	409	0.910 (0.902–0.919)
67	3389	456	0.881 (0.871–0.892)
Total	18 825	1358	0.933 (0.929–0.936)

Abbreviations: CI=confidence interval; FP=false positive; TN=true negative.

a TN: No. of men with negative screening result (serum PSA<3.0 ng ml^−1^ or PSA 3.0–3.9 ng ml^−1^ with a negative ancillary examination (benign finding at digital rectal examination or free/total PSA ratio⩾0.16)).

b FP: No. of men with positive screening result (serum PSA<4.0 ng ml^−1^ or PSA 3.0–3.9 ng ml^−1^ with a positive ancillary examination (suspicious finding at digital rectal examination or free/total PSA ratio<0.16)) minus number of screen-detected cancers. Note: men refusing biopsy (*N*=102) excluded.

c Specificity: TN/(TN+FP).

**Table 3:** Number of men by screening result and prostate cancer diagnosis in the second screening round of the Finnish prostate cancer screening trial

	**Prostate cancer diagnosis**		
**Screening result**	**Yes**	**No**	**Total**
Positive	583^a^	1573	2156^b^
Negative	45^c^	16 264	16 309
Total	628	17 837	18 465^b^

a Screen-detected prostate cancer.

b A total of 147 screen-positive men failed to undergo biopsy and were excluded.

c Interval cancer among screen-negative men.

**Table 4:** The frequency of TN and FP screening findings by age in the second screening round of the Finnish prostate cancer screening trial

**Age**	**TN^a^**	**FP^b^**	**Specificity (95% CI)^c^**
59	5700	322	0.947 (0.941–0.952)
63	4464	434	0.911 (0.903–0.919)
67	3426	395	0.897 (0.887–0.906)
71	2719	422	0.866 (0.854–0.878)
Total	16 309	1573	0.912 (0.908–0.916)

Abbreviations: CI=confidence interval; FP=false positive; TN=true negative.

a TN: No. of men with negative screening result (serum PSA<3.0 ng ml^−1^ or PSA 3.0–3.9 ng ml^−1^ with a negative ancillary examination (benign finding at digital rectal examination or free/total PSA ratio⩾0.16)).

b FP: No. of men with positive screening result (serum PSA<4.0 ng ml^−1^ or PSA 3.0–3.9 ng ml^−1^ with a positive ancillary examination (suspicious finding at digital rectal examination or free/total PSA ratio<0.16)) minus number of screen-detected cancers (147 men without biopsy excluded).

c Specificity: TN/(TN+FP).
